# Deafferentation of the Superior Colliculus Abolishes Spatial Summation of Redundant Visual Signals

**DOI:** 10.3389/fnsys.2017.00009

**Published:** 2017-02-24

**Authors:** Martijn van Koningsbruggen, Kristin Koller, Robert D. Rafal

**Affiliations:** ^1^Wolfson Centre for Clinical and Cognitive Neuroscience, School of Psychology, Bangor UniversityBangor, UK; ^2^Department of Psychological and Brain Sciences, University of Delaware, NewarkDE, USA

**Keywords:** superior colliculus, brachium of the superior colliculus, neural summation, pulvinar, DTI tractography

## Abstract

Two visual signals appearing simultaneously are detected more rapidly than either signal appearing alone. Part of this redundant target effect (RTE) can be attributed to neural summation that has been proposed to occur in the superior colliculus (SC). We report direct evidence in two neurological patients for neural summation in the SC, and that it is mediated by afferent visual information transmitted through its brachium. The RTE was abolished in one patient with a hemorrhage involving the right posterior thalamus that damaged part of the SC and that disrupted its brachium; and in another patient in whom the SC appeared intact but deafferented due to traumatic avulsion of its brachium. In addition reaction time for unilateral targets in the contralesional field was slowed in both patients, providing the first evidence that visual afferents to the SC contribute to the efficiency of target detection.

## Introduction

Two visual signals appearing simultaneously are detected more rapidly than either signal appearing alone (Hershenson, 1962; Raab, 1962). Part of this redundant target effect (RTE) can be accounted for stochastically by a ‘horse-race’ model. That is, if two stimuli are processed in parallel and independent channels for which processing speed varies randomly from trial to trial, on each presentation the fastest stimulus wins the race and reaches detection threshold to trigger the response; therefore, on average, two stimuli are more likely to yield a faster response than the average response time to one stimulus processed in either of the two channels.

However, analyses of cumulative frequency distributions have shown there to be an additional contribution to the RTE that cannot be accounted for by a race horse model between two independent channels. Miller (1982) proposed a ‘co-activation’ model in which the two signals are summed in an activation pool. Miniussi et al. (1998) showed that redundant targets produced shorter latencies for P1 and N1 event-related brain potentials, indicating that neural summation occurs early in the visual pathway. Experiments in hemianopic patients have also shown that the RTE cannot be fully accounted for by a horse race to reach detection threshold, since a RTE can be manifest even in the absence of phenomenal awareness of one of the two stimuli (Marzi et al., 1986; Tomaiuolo, 1997; de Gelder et al., 2001; Leh et al., 2006a,b). Indeed, in hemispherectomy patients a redundant target in the blind field not only generates a RTE, the RTE is augmented when the redundant stimulus completes a gestalt pattern (Georgy et al., 2016). These observations in hemianopics suggest that neural summation occurs subcortically and is not dependent on primary visual cortex.

The cumulative indirect evidence has implicated superior colliculus (SC) as the substrate for the neural summation that contributes to the RTE. However, direct evidence has not yet been reported. The current research investigates the neural pathway that transmits the visual signals that are integrated in the SC. The SC consists of superficial and deep layers. The deep layers of the colliculus receive afferents, both direct and via the basal ganglia, from oculomotor cortex (Hikosaka and Wurtz, 1983; Pare and Wurtz, 1997; Sommer and Wurtz, 2000). Visual afferents to the superficial layers of the colliculus, however, are transmitted from both the retina (via the retino-tectal tract) and from early visual cortex (Fries, 1984), via the brachium of the SC.

Since neural summation effects have been demonstrated in neurological patients lacking a visual cortex, the visual signals summated in the colliculus could be transmitted directly from the retina to superficial layers of the colliculus via the retino-tectal tract. If this is the case, neural summation could be abolished by lesions of the brachium of the SC.

Lesions that disrupt the brachium of the SC are very rare. Here we report single case studies of two patients with unilateral subcortical lesions that deafferented the SC from visual input, and who did not manifest a RTE. One patient had a lesion damaging both the pulvinar nucleus of the thalamus, the rostral SC, and its brachium. In the other patient, with damage to the dorsal midbrain from a traumatic brain injury, the SC appeared to be intact, but its brachium was disrupted.

## Materials and Methods

### Participants

Two neurological patients, each with her own age matched control group, were tested in an experiment to measure the RTE.

**FIGURE 1:**
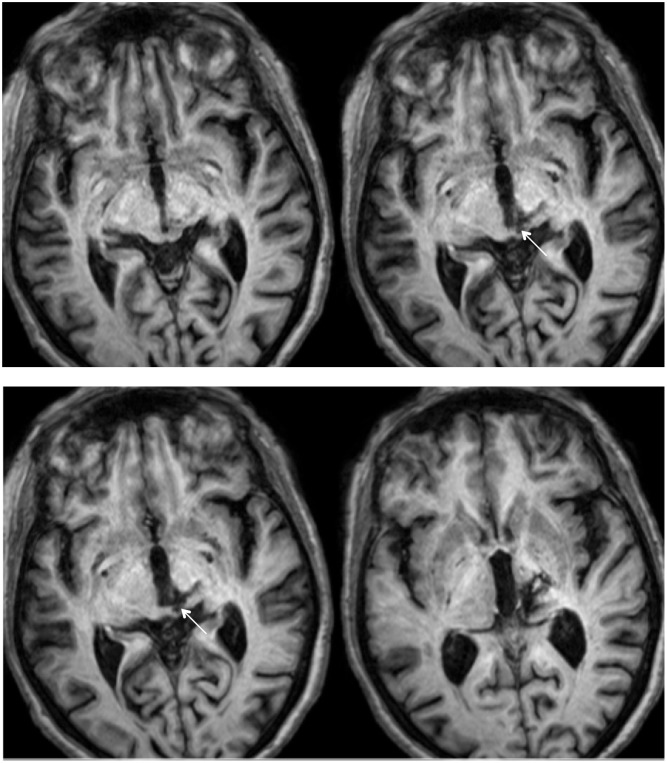
**High resolution (0.7 mm^3^) T1-weighted MRI of patient RE.** Axial slices from ventral (Top left) to dorsal (Bottom right) showing the lesion in the right dorsal midbrain and thalamus. Extension of the lesion into the rostral superior colliculus (SC) shown by white arrows.

**FIGURE 2:**
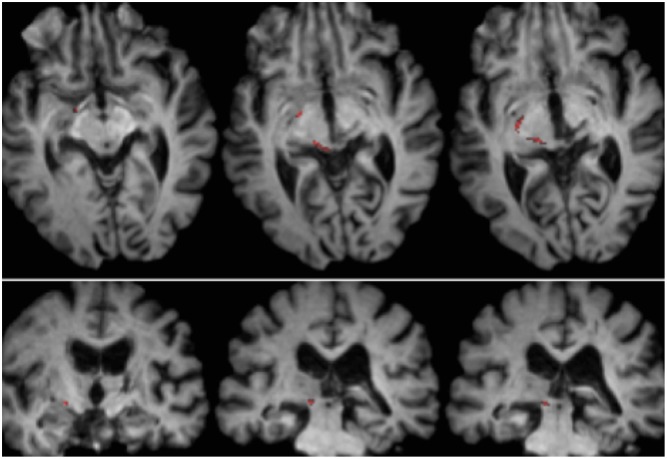
**Tractography demonstrating the retinotectal tract in patient RE co-registered to T1-weighted axial slices (Top from ventral to dorsal) and coronal slices (Bottom from anterior to posterior).** The course of the retinotectal tract is shown in the left hemisphere (red). No streamline was traced in the right hemisphere and, by comparison with the intact hemisphere it can be seen that the lesion destroyed the brachium of the SC in the right hemisphere. The streamline shown in red was generated with probabilistic tractography using FSL FDT (FMRIB Diffusion Toolbox; Behrens et al., 2003, Behrens et al., 2007; http://fsl.fmrib.ox.ac.uk/fsl/fslwiki). Diffusion-weighted echo-planar magnetic resonance images were acquired at 1.5 mm^3^ resolution with 32 isotropically distributed diffusion-encoding directions (*b* = 800) and a baseline (*b* = 0). Repetition time = 2 s, and echo time = 35 ms. For the probabilistic tractography, we manually marked the starting region (i.e., drew seed masks) on the optic tract of each hemisphere just posterior to the chiasm and a target region (target mask) on the SC.

RE was 71 years old at the time of testing. A posterior thalamic hemorrhage 3 years earlier destroyed the medial pulvinar, ventro-lateral thalamic nuclei and the posterior limb of the internal capsule with damage extending into the dorsal midbrain including the pretectum and rostral SC (**Figure 1**) Probabilistic DTI tractography (Behrens et al., 2003, Behrens et al., 2007) confirmed that the lesion destroyed the brachium of the SC (**Figure 2**). RE was paralyzed on the left side of her body with loss of sensation in the left face and arm. She had abnormal eye movements including lid retraction, paralyzed vertical eye movements, and impaired convergence, which were a consequence of lesion extension into the dorsal midbrain.

**FIGURE 3:**
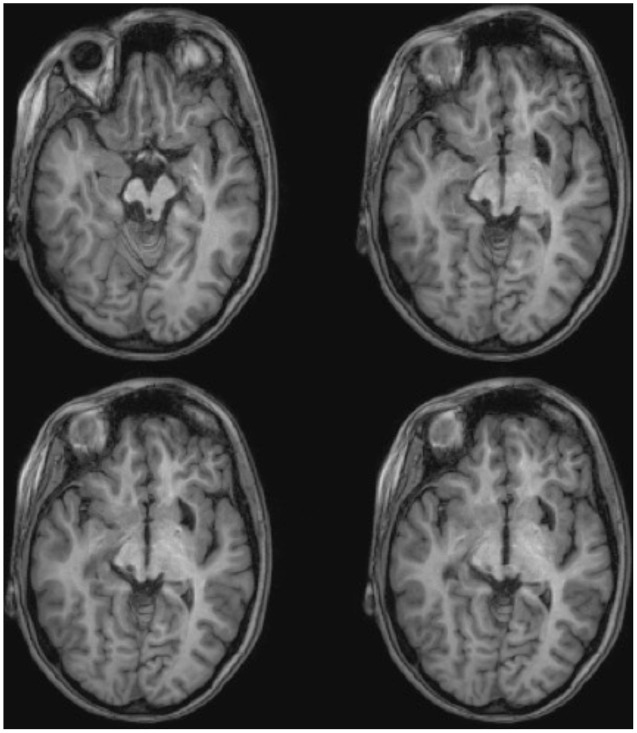
**High resolution (0.7 mm^3^) T1-weighted MRI of patient ML.** Axial slices from ventral (Top left) to dorsal (Bottom right) showing damage to the left inferior colliculus and mesencephalic tegmentum ventral and lateral to the SC.

**FIGURE 4:**
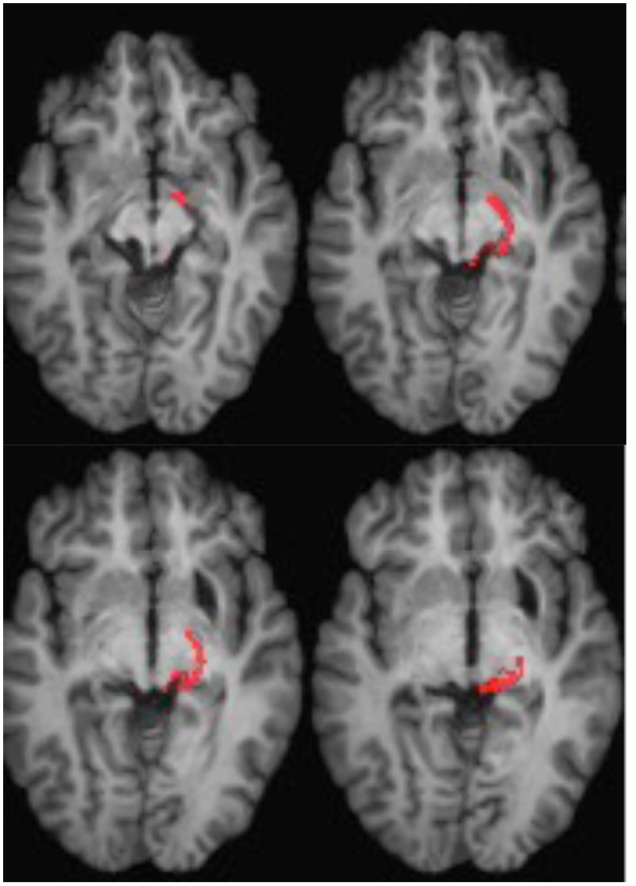
**DTI tractography in patient ML showing the retino-tectal tract in the right hemisphere (red) co-registered to T1-weighted MR images in the axial plane from ventral (Top left) to dorsal (Bottom right).** Diffusion-weighted echo-planar magnetic resonance images were acquired at 2 mm^3^ resolution with 63 isotropically distributed diffusion-encoding directions (*b* = 1000) and a baseline (*b* = 0). Repetition time = 2 s, and echo time = 35 ms. Tractography was implemented as described in **Figure 2**.

ML was 23 years old at the time of testing. She had sustained a severe traumatic brain injury in a road traffic accident 7 years earlier resulting in diffuse axonal injury with hemorrhage into the right putamen and dorsal midbrain. Details of her history, neurological examination, and neuroimaging findings have been reported by (Poliva et al., 2015). Although left was some motor impairments, including spasticity and ataxia, she had regained independence. Her chief disability was severe auditory agnosia due to damage to brain stem auditory pathways including the left inferior colliculus. Visual acuity and visual fields were unimpaired and there was no visual neglect or extinction. Oculomotor signs of midbrain dysfunction included macro-square wave jerks and convergence spasm on vertical gaze (downward more than upward.) There was no ptosis or pupillary abnormality. High resolution MRI showed a cystic cavity in the right putamen at the site of her previous hemorrhage, and small periventricular lesion on the right lower pons, in the region of the inferior cerebellar peduncle (**Figure 3**). She had nearly complete avulsion of the left inferior colliculus, sparing only its most medial and caudal parts. Damage extended into the lateral pretectum and midbrain tegmentum destroying the brachia of the superior and inferior colliculi, and ventrally into the red nucleus. Probabilistic DTI tractography confirmed that the lesion destroyed the brachium of the SC (**Figure 4**).

Neurologically healthy control subject for patient RE included 11 older adults (six women and five men, mean age: 68.5, range 59–73). They were recruited from the Bangor University community participant panel. Participants had no known neurological, psychological, psychiatric, or cognitive impairments and all participants had normal/corrected to normal vision. Participants received payment of £6 for their participation.

Neurologically healthy control subjects for patient ML included 11 Bangor University undergraduates and postgrads ranging in age from 18–26 (seven women and four men).

### Apparatus and Viewing Conditions

Presentation^®^ programming software running on a PC computer recorded reaction time (RT) responses and generated stimuli that were presented on a Dell monitor (12.5″ × 25″, refresh rate 60 Hz.) placed at eye level 57 cm in front of participants with binocular viewing in dimmed light conditions. A chin and headrest was used to secure head stability. Participant responses were recorded via spacebar key-press on a keyboard.

### Stimuli

The experimental stimuli consisted of unfilled white marker squares (1 cm × 1 cm) positioned 8° to left and right of a small fixation box (0.2 cm × 0.2 cm) at the center of the screen. Target stimuli consisted of filling in of one or both peripheral marker boxes to produce a solid white square. All stimuli were white presented on a gray background.

**FIGURE 5:**
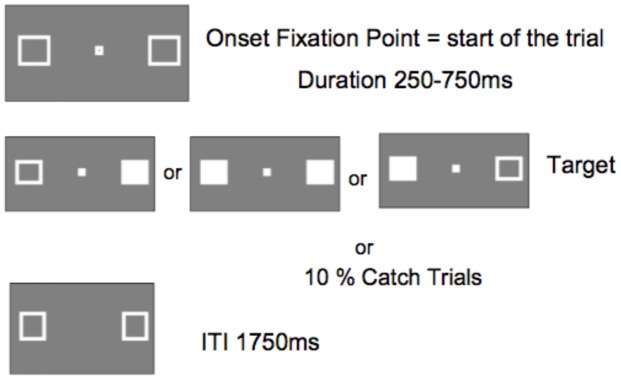
**Display sequence of a single trial (ITI, inter-trial interval between participant response an onset of fixation point starting the next trial)**.

**Figure 5** shows the sequence of a single trial. The marker boxes remained visible throughout the experiment. After an inter-trial interval of 1750 ms, the fixation box appeared to start the trial. After a random interval ranging from 250 to 750 ms (in 25 ms increments), a target requiring a simple key press response on the keyboard space bar, appeared at the location of one or both marker boxes. The target was a filled white square generated by filling in one or both maker boxes. Randomly and with equal probability, targets appeared on the left, the right, or both simultaneously. No target appeared on catch trials (10% of trials).

### Procedure

Each participant was tested in a single session. After reading instructions on-screen, participants were shown examples of all the stimuli that were presented in the task (the fixation box and each of the three target presentations) and was asked to confirm recognition of each target via space-bar key press. The instructions were to maintain fixation on the central box for the duration of each trial, and to respond as quickly as possible by pressing the keyboard space bar with the right hand as soon as a target appeared.

Once the participant confirmed verbally and via key-press that all instructions were understood, presentation of practice trials (10 of each target presentation condition and 3 target-absent ‘catch’ trials) proceeded. On-screen feedback was presented during practice trial completion including the following statements: (1) “Correct,” (2) “Try to respond faster!” and (3) “Only respond if you see a target.” Once the practice session was completed and both the participant and experimenter were confident that the task instructions were understood correctly, the participant was invited to begin the experiment. The first 30 trials of each experimental block were excluded as practice trials. Each block consisted of a total of 233 trials: 70 right, 70 left, 70 both, and 23 ‘catch’ trials. A short break was given half way through the block. Patient ML and the younger controls were tested in a single block. Because of the variability related to Patient RE’s age and condition, she was tested on two blocks on separate days; and her age-matched control participants were also tested on two sessions on a single day.

### Analyses

For each control group, median RT for each participant in each condition (left target, right target, bilateral targets) was calculated after excluding trials following catch trials and those with RTs of <100 ms or >800 ms. A paired sample *t*-test was done to confirm that there was no asymmetry for responses to right and left unilateral targets, and a mean RT for unilateral targets was calculated. RTE was computed by subtracting RTs for bilateral trials from RTs for unilateral trials. A paired sample *t*-test compared unilateral with bilateral to establish whether the RTE was statistically reliable.

For each patient, after excluding trials following catch trials and those with RTs of <100 ms or >800 ms, a paired sample *t*-test compared median RTs for contralateral and ipsilateral unilateral targets. As reported below, RTs for responses to contralesional targets were longer than for ipsilesional unilateral targets. To determine whether the presence of a contralesional target engendered a statistically reliable RTE, a paired samples *t*-test compared median RTs for bilateral target trials with unilateral ipsilesional target trials.

To test whether the RTE was reliably smaller for each patient than for their respective control group, RTE was computed for each control participant and the upper and lower bounds of the 95% confidence intervals (CI) were calculated. The *Z*-score for each patient’s RTE was computed relative to their control group and tested for statistical reliability with Crawford’s *t*-test.

*P* values for paired sample *t*-tests were reported for two tails and for Crawford *t*, one tailed.

## Results

### Patient RE

**FIGURE 6:**
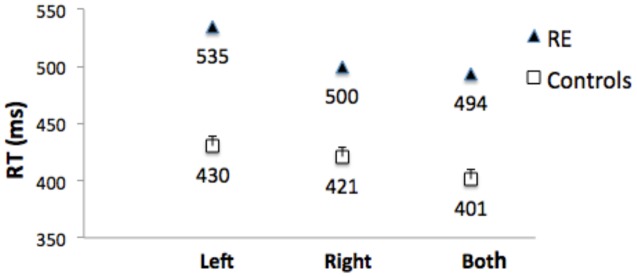
**Median RTs for Patient RE, and mean of the median RTs for control group participants for targets appearing in the left visual field (‘Left’), right visual field (‘Right’), and simultaneously in both visual fields (‘Both’).** Error bars for control participants denote standard errors.

Control Participants: Mean of the median RTs did not differ for left and right responses (*t*[1,10] = 0.6.) **Figure 6** shows that RTs were shorter (the RTE) for bilateral targets than for either left (*t*[1,10] = 6.8, *p* < 0.001, or right (*t*1,10) = 3.8, *p* = 0.003) unilateral targets. Median RT for bilateral target trials was subtracted from the mean of the median RT for unilateral targets to compute a mean RTE for each control participant. The control group mean RTE was 24.3 ms (SEM = 2.75).

Patient RE’s median RTs were longer than controls (**Figure 6**), and were above the upper bound of the 95% CI of the control group mean RTs for all three conditions (left = 457 ms; right = 449 ms; both = 426 ms). RTs to unilateral targets in the contralesional (left) field were longer than for unilateral targets in the ipsilesional (right) field (*t*[1,233] = 6.0, *p* < 0.001).

Importantly, there was no RTE: Median RT for bilateral targets (500 ms) was not shorter than for unilateral targets in the ipsilesional (right) visual field (494 ms; *t*[1,244] = 1.141), *p* = ns (**Figure 6**). **Table 1** (top) shows that the RTE calculated for patient RE was significantly less than for the RTE for her control group.

**Table 1:** Redundant target effect for patient RE (top) and ML (bottom), and mean RTE for their respective control groups with 95% confidence intervals (CI), *Z*-scores and Crawford *t-*test comparing each patient with her respective control group.

Control RTE 95% CI (ms)		Control mean RTE (ms)	Patient RE RTE (ms)	*Z*-score	Crawford *t*
Upper bound	30.4				
		24.3	6	-3.65	-2.1, *p* < 0.05
Lower bound	18.1				
Upper bound	18.4				
		33.0	9	-4.24	-3.6, *p* < 0.005
Lower bound	10.3				


*The RTE for patients was calculated by subtracting median RT for bilateral targets from median RT for ipsilesional unilateral target and was not significantly different from zero for either patient.*

### Patient ML

**FIGURE 7:**
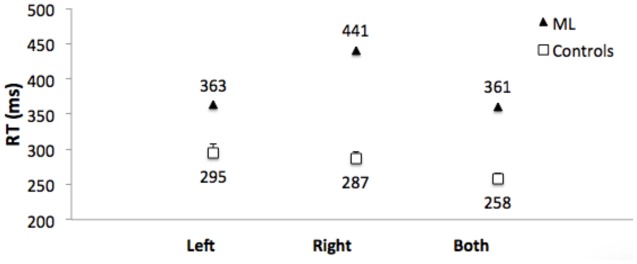
**Median RTs for Patient ML, and mean of the median RTs for control group participants for targets appearing in the left visual field, right visual field, and simultaneously in both visual fields.** Error bars for control participants denote standard errors.

Control Participants: Mean of the median RTs did not differ for responses to left and right targets (*t*[1,11] = 0.86. **Figure 7** shows that RTs were shorter (the RTE) for bilateral targets than for either left (*t*[1,11] = 4.7, *p* = 0.001, or right (*t*1,11) = 7.8, *p* < 0.001) unilateral targets.

Median RT for bilateral target trials was subtracted from the mean of the median RT for left and right unilateral targets to compute a RTE for each control participant.

The group mean RTE was 33 ms (SEM = 6.65).

Patient ML’s median RTs were longer than controls (**Figure 7**), and were above the upper bound of the 95% CI of the control group mean RTs for all three conditions (left = 322 ms; right = 307 ms; both = 277 ms). RTs to unilateral targets in the right (contralesional) field were longer than for unilateral targets in the left (ipsilesional) field (*t*[1,116] = 4.9, *p* = 0.001). There was no RTE. RTs for bilateral targets (361 ms) were not shorter than for unilateral targets in the ipsilesional (left) visual field (363 ms). **Table 1** (bottom) shows that the RTE calculated for patient ML was significantly less than the RTE for her control group.

## Discussion

Simple RTs were measured to detect single targets in either contralesional or ipsilesional field, and bilateral targets, in two patients in whom the brachium of the SC was damaged in one hemisphere. In one of the patients (a young woman) the damage was in the left hemisphere and was due to traumatic brain injury; and, while the lesion involved the inferior colliculus and extended into the midbrain tegmentum, damage to the SC *per se* was not evident with high-resolution neuroimaging. In the other patient (an older woman) the damage was in the right hemisphere and was caused by hypertensive hemorrhage that also caused extensive damage to the medial pulvinar and lateral thalamus and extended into the dorsal midbrain including the rostral SC. In both patients simple RT to detect visual targets in the contralesional field was slowed compared to the ipsilesional field. Neither patient showed a spatial summation effect (RTE) for target detection when stimuli were presented to both visual fields.

These observations shed light on the role of the SC in target detection and, more specifically, the role of visual afferents to the SC. Posner et al. (1980) operationally defined detection as being evidenced by the ability to make an *arbitrary* response to a visual signal (e.g., a simple key press with a finger, as in the current experiment). They posited that detection requires an allocation of attention to select the target for processing in a limited capacity system that prioritizes it to be acted upon.

Song et al. (2011) showed that that inactivation of monkey SC causes striking target selection deficits that cannot be readily explained as a simple impairment in visual perception or motor execution, and suggested that it contributes to a more general purpose priority map. In that experiment monkeys were presented with two stimuli in opposite visual fields and had to indicate, with a reaching response, which appeared first. One monkey was trained to reach toward the stimulus that appeared first; and one monkey was trained to reach toward the target that appeared second. In both cases, there was a strong bias against selecting the target that was in the visual field contralateral to the lesion. Since the same bias was seen in the monkey who reached to the second target that appeared, the bias could not be attributable to a delay in perceiving the contralesional target. Collicular inactivation did not cause an impairment in the perceptual judgment of which stimulus appeared first. Rather, stimuli in the visual field contralateral to the inactivated colliculus were disadvantaged in being prioritized for action.

Zhaoping (2016) has recently highlighted a distinction between neural saliency maps and priority maps, and argued for an evolutionary migration of a perceptual saliency map from the optic tectum/SC to primary visual cortex. She proposed that salience signals can be transmitted to a priority map in the SC without projecting the feature tuning property.

The brachium of the SC transmits afferent visual signals to the superficial layers of the colliculus from the retina via the retino-tectal tract; but the majority of visual afferent fibers transmitted through the brachium are projections from primary visual cortex. If the brachium is disrupted, salience signals from primary visual cortex can be relayed to the colliculus via the frontal eye fields. But in the absence of direct projections to the superficial layers of the colliculus from the retina or from visual cortex, as we presume to be the case in the patients with brachium lesions, it takes longer for the collicular priority map to be activated, resulting in slower responses to contralesional visual signals.

As noted in the introduction, the contribution to the RTE that is based upon a probabilistic race horse model is dependent upon detection of both targets; and on a race in which the efficiency of independent channels in which the two targets are transmitted are equivalent, such that the outcome of the race to reach detection threshold is random from trial to trial. Since the detection threshold for contralesional signals is higher in the patients reported here, redundant targets could not benefit based on a stochastic horse race; that is, the outcome of the race between ipsilesional and contralesional signals is not random – contralesional signals are more likely to lose the race than to win it.

Nevertheless, research in hemianopic patients has shown that there is also a neural summation component that contributes to the RTE which does not depend upon detection of both targets (Marzi et al., 1986; Tomaiuolo, 1997; de Gelder et al., 2001). Since commissurotomized patients have been shown to have an RTE(Marzi et al., 1986; Reuter-Lorenz et al., 1995), it has been argued that interhemispheric integration of signals across the vertical meridian must occur subcortically.

Savazzi and Marzi (2004) showed that neural summation did not occur with short wave length chromatic target stimuli in either neurologically intact people or split brain patients. Leh et al. (2006b) reported that, while a RTE did occur with achromatic stimuli in some hemianopic hemispherectomized patients, there was no RTE in these patients when short wave length chromatic stimuli were used as targets. Short wave length (i.e., purple) stimuli activate only S-cones in the retina. Retinal ganglion cells that receive input from S-cones do not project either directly to the SC, or to magnocellular layers of the lateral geniculate nucleus (De Monasterio, 1978). Since projections from primary visual cortex to the SC through the brachium of the SC relay visual only visual signals from magnocellular geniculostriate afferents (Schiller et al., 1979), the failure of S-cone stimuli to engender a RTE suggests that neural summation occurs in the SC and is dependent upon visual afferents transmitted through its brachium.

There is, thus, converging evidence in split brain patients, hemianopic patients with blindsight, and from experiments using short wave length stimuli, that neural summation occurs in the SC. Nevertheless, while short wave length stimuli do not activate retinal ganglion cells that project to the SC via the brachium, it cannot be concluded that the colliculus is entirely blind to such stimuli. Single unit recordings in monkey SC have demonstrated that short wave length stimuli do activate responses in superficial layers of the colliculus. Furthermore, tractography has demonstrated, in those hemispherectomized patients with a RTE (i.e., blindsight), but not in those patients who did not show evidence of blindsight, that the SC had connection to the intact hemisphere (Leh et al., 2006a).

The findings of the current investigation, thus, provide the first direct evidence that spatial summation occurs in the SC that is dependent on transmission of visual signals through its brachium.

## Ethics Statement

This study was carried out in accordance with the guidelines and with approval of the protocol of the NHS Ethics Committee, Bangor, UK, and the School of Psychology Ethics Committee of Bangor University, UK. Oral and written informed consent was obtained from all participants in accordance with the Declaration of Helsinki.

## Author Contributions

MvK: Conceived the research, designed and programmed the experiment, collected and analyzed patient data and contributed to writing the manuscript. KK: Collected control data, reviewed relevant literature, analyzed control data and contributed to writing the manuscript. RR: Recruited patients and analyzed neuroimaging studies including virtual dissection with DTI tractography, prepared patient case histories and contributed to writing the manuscript.

## Conflict of Interest Statement

The authors declare that the research was conducted in the absence of any commercial or financial relationships that could be construed as a potential conflict of interest.
